# The Role of Dopamine in the Collective Regulation of Foraging in Harvester Ants

**DOI:** 10.1016/j.isci.2018.09.001

**Published:** 2018-09-27

**Authors:** Daniel A. Friedman, Anna Pilko, Dorota Skowronska-Krawczyk, Karolina Krasinska, Jacqueline W. Parker, Jay Hirsh, Deborah M. Gordon

**Affiliations:** 1 Department of Biology, Stanford University, Stanford, CA 94305, USA; 2 Department of Chemistry and Biochemistry and the Institute for Quantitative and Computational Biosciences (QCB), University of California, Los Angeles, Los Angeles, CA 90095, USA; 3 Shiley Eye Institute, Richard C. Atkinson Lab for Regenerative Ophthalmology, Department of Ophthalmology, University of California, San Diego, La Jolla, CA 92093, USA; 4 Stanford University Mass Spectrometry, Stanford University, Stanford, CA 94305, USA; 5 Department of Biology, University of Virginia, Charlottesville, Charlottesville, VA 22904, USA

**Keywords:** Entomology, Neuroscience, Molecular Neuroscience

## Abstract

Colonies of the red harvester ant (*Pogonomyrmex barbatus*) differ in how they regulate collective foraging activity in response to changes in humidity. We used transcriptomic, physiological, and pharmacological experiments to investigate the molecular basis of this ecologically important variation in collective behavior among colonies. RNA sequencing of forager brain tissue showed an association between colony foraging activity and differential expression of transcripts related to biogenic amine and neurohormonal metabolism and signaling. In field experiments, pharmacological increases in forager brain dopamine titer caused significant increases in foraging activity. Colonies that were naturally most sensitive to humidity were significantly more responsive to the stimulatory effect of exogenous dopamine. In addition, forager brain tissue significantly varied among colonies in biogenic amine content. Neurophysiological variation among colonies associated with individual forager sensitivity to humidity may reflect the heritable molecular variation on which natural selection acts to shape the collective regulation of foraging.

## Highlights

• Red harvester ant colonies vary in how they adjust foraging effort in dry conditions
• Colonies that differ in behavior significantly differ in forager brain transcriptome
• Pharmacological increases of dopamine increased foraging in field experiments
• Foragers from colonies more sensitive to humidity were more stimulated by dopamine

## Graphical Abstract

**Figure:**
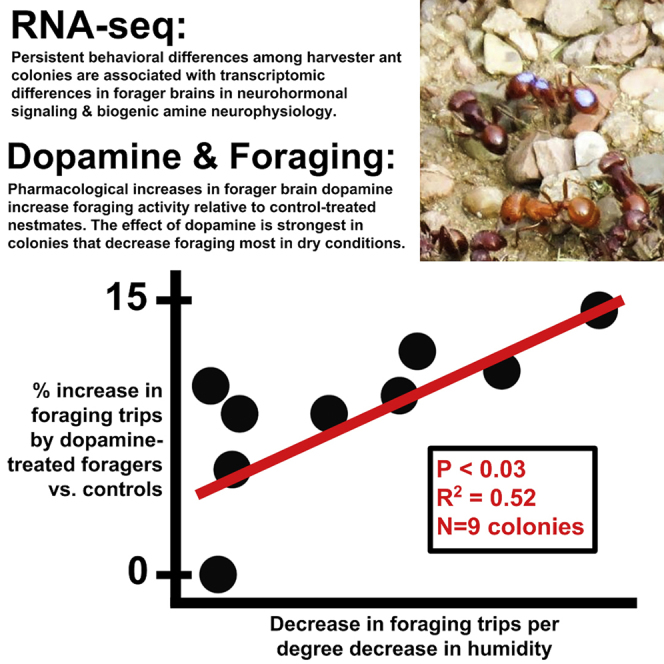

## Introduction

Many biological systems, from brains to insect colonies, are regulated by distributed processes based on local interactions. Variation among groups in collective behavior (Pruitt et al., 2017) can arise from differences between groups in individual response to local interactions or from differences in group composition (Bengston and Jandt, 2014, Jandt et al., 2014). In social insects, such as ants and honeybees, collective behavior is regulated through olfactory interactions among workers (Dornhaus and Franks, 2008, Feinerman and Korman, 2017, Gordon, 1996, Gordon, 2010, Gordon and Mehdiabadi, 1999). Ants are a globally distributed clade of social insect species (Gibb et al., 2017, Parr et al., 2017, Ward, 2014), and ecological factors shape the evolution of ant collective behavior (Gordon, 2013, Gordon, 2014, Lanan, 2014). Rapid advances in high-throughput sequencing technologies are providing insight into the genomic, transcriptomic, and epigenomic differences among social insect species (Boomsma et al., 2017, Favreau et al., 2018, Toth and Rehan, 2017). Molecular studies have characterized various mechanistic aspects of division of labor among workers within social insect colonies (Friedman and Gordon, 2016, Kamhi and Traniello, 2013, Linksvayer, 2015, Simola et al., 2016), building on a long history of diverse research into social insect behavior (Detrain and Deneubourg, 2006, Gordon, 1992, Hölldobler and Wilson, 2009, Seeley, 2010, von Frisch, 1974). However, much less is known about the molecular variation among social insect colonies associated with heritable variation in collective behavior (Bengston and Jandt, 2014, Jandt et al., 2014, Jandt and Gordon, 2016).

Across ant and bee species, variation among nestmates in reproductive status and behavioral performance are associated with tissue-specific physiological and transcriptomic differences (Chandra et al., 2018, Gordon, 2016a, Jeanne, 2016, Johnson and Linksvayer, 2010, Toth and Dolezal, 2017). Worker brain biogenic amine and neurohormonal signaling pathways are especially important in regulating the foraging activity of social (Friedman and Gordon, 2016, Gospocic et al., 2017, Kamhi and Traniello, 2013, Yan et al., 2014) and solitary insects (Kamhi et al., 2017, Perry and Barron, 2013, Waddell, 2013). Changes in brain biogenic amine content influence individual worker behavior by altering their sensitivity to certain stimuli such as foraging cues (Bubak et al., 2016, Kamhi and Traniello, 2013, Muscedere et al., 2012, Scheiner et al., 2017). Natural variation among nestmates in sensitivity to stimuli can be adaptive for colony function, for example, by allowing for dynamic task allocation (Gordon, 1989, Gordon and Mehdiabadi, 1999). Dopamine appears to be central to the regulation of individual foraging activity in social insects (Friedman and Gordon, 2016, Kamhi and Traniello, 2013) and other animals (Barron et al., 2010, Calhoun et al., 2015, Clark and Dagher, 2014, Friston et al., 2012, Perry and Barron, 2013, Waddell, 2013). In honeybees, brain dopamine titers are higher in foragers than in non-foragers (Tedjakumala et al., 2017, Wagener-Hulme et al., 1999), and pharmacological increases in brain dopaminergic signaling increase foraging activity (Mustard et al., 2012, Perry et al., 2016, Scheiner et al., 2017, Søvik et al., 2014, Søvik et al., 2015b). In many ant species, foragers have higher brain dopamine titer than workers of other task group (Kamhi et al., 2017, Seid and Traniello, 2005, Smith et al., 2013), although in some species this pattern appears to be reversed (Penick et al., 2014). Laboratory pharmacological studies in carpenter ants confirm a role for dopaminergic signaling in the regulation of individual foraging activity (Entler et al., 2016). In ants, dopamine is also involved in trophallaxis (Wada-Katsumata et al., 2011) and reproduction (Okada et al., 2015, Penick et al., 2014). Recent pharmacological experiments in the field have been used to examine ant behavior (Malé et al., 2017), but not the biogenic amine neurophysiology of foraging.

Colonies of the red harvester ant, *Pogonomyrmex barbatus*, forage in the desert for seeds that provide both food and water. Foragers lose water while out in the desert sun, and the rate of water loss is higher in dry conditions (Lighton and Bartholomew, 1988, Lighton and Feener, 1989). To manage the tradeoff between food accumulation and water loss, colonies adjust foraging activity to changes in ambient conditions, especially humidity (Gordon, 2013, Gordon et al., 2013, Prabhakar et al., 2012). Colony foraging activity is regulated in a distributed fashion by brief olfactory interactions when one ant assesses the cuticular hydrocarbons of the other (Greene et al., 2013, Greene and Gordon, 2003): an outgoing forager is stimulated to leave the nest by its rate of interaction with incoming foragers with food (Davidson et al., 2016, Greene et al., 2013, Pinter-Wollman et al., 2013, Pless et al., 2015). Since a forager continues to search until it finds a seed, the rate of forager return is related to food availability (Gordon, 1991). Colonies of *P. barbatus* significantly vary in how strongly they reduce foraging activity in dry conditions (Gordon, 1991, Gordon, 2013, Gordon et al., 2011, Gordon et al., 2013), meaning that colonies differ in their sensitivity to humidity. These behavioral differences among colonies of *P. barbatus* persist year after year despite total worker turnover (Gordon, 1991, Gordon, 2013, Gordon et al., 2011, Gordon and Hölldobler, 1987), and daughter colonies resemble their mothers in the thresholds for dry conditions that lead them to reduce foraging (Gordon, 2013). This variation among colonies of *P. barbatus* in foraging behavior is ecologically important and associated with differences in colony lifetime reproductive success (Gordon, 2013, Ingram et al., 2013). Colony differences in collective behavior could be due to stable colony differences in how foragers adjust their sensitivity to interactions in dry conditions (Davidson et al., 2016, Pagliara et al., 2018).

Here we examine the neurophysiological basis of variation among red harvester ant colonies in how they regulate their collective foraging behavior. Because differences among *P. barbatus* colonies in sensitivity to humidity persist year after year, it appears that successive cohorts of nestmate foragers inherit genetic or epigenetic factors, which bias their foraging activity in dry conditions. Molecular variation among foragers from different colonies may lead to differences in individual forager decisions about whether to forage in dry conditions. This would produce the observed variation among colonies in the collective regulation in foraging. The specific molecular mechanisms that might underlie forager sensitivity to humidity are not known.

To investigate this, we first used RNA sequencing (RNA-seq) to assess transcriptomic differences in forager brain tissue between 2 sets of colonies that naturally varied in how strongly they reduced foraging activity in dry conditions. Patterns of transcript differential- and co-expression between the 2 sets of colonies included significant changes in biogenic amine and neurohormonal pathways. To test the role of dopamine signaling in the regulation of individual foraging activity, we manipulated the brain dopamine titer of foragers in field experiments during 2 consecutive years. Foragers with increased brain dopamine titer made significantly more foraging trips than control-treated nestmates, and foragers treated with a metabolic inhibitor of dopamine synthesis significantly decreased foraging activity relative to control-treated nestmates. In the set of 9 colonies used in pharmacological experiments, we also characterized natural patterns of behavioral variation and forager brain biogenic amine content. Colonies that were naturally more sensitive to humidity tended to be more responsive to the stimulatory effect of exogenous dopamine. This suggests that a forager's decision whether to leave the nest on its next trip may be influenced by dopamine, so that variation among colonies in the regulation of foraging in response to conditions may be due to differences among colonies in forager biogenic amine neurophysiology.

## Results

### Forager Brain Transcriptomic Differences Are Associated with Differences in Colony Behavior

**Figure 1:**
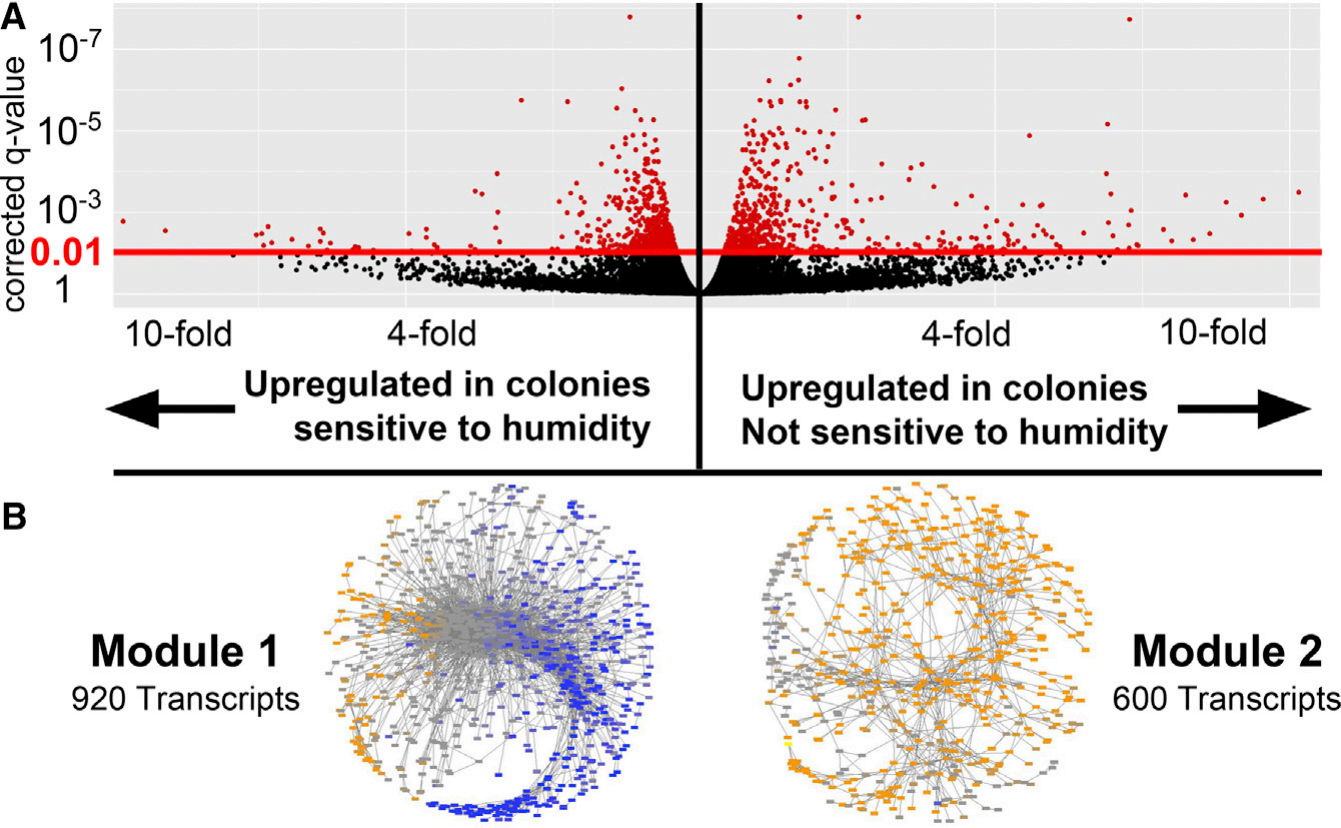
Forager Brain Transcriptomic Differences Are Associated with Differences in Colony Behavior (A) Volcano plot representing forager brain gene expression differences between groups of colonies that vary in their sensitivity to humidity (see Transparent Methods 1A). Red transcripts are significantly differentially expressed at FDR-corrected q-value < 0.01. (B) Transcript co-expression graph. Nodes are transcripts that are colored according to mean fold-change between the 2 sets of colonies. Edges connecting nodes represent correlated expression levels across all 18 libraries. Blue nodes are transcripts upregulated in foragers from colonies that strongly reduce foraging on dry days. Orange nodes are transcripts upregulated in foragers from colonies that do not strongly reduce foraging on dry days. Gray nodes are transcripts that were evenly expressed between the 2 groups of colonies.

Forager brain transcriptomes differed between 2 sets of *P. barbatus* colonies that differed in how strongly they reduced foraging activity in dry conditions (see Transparent Methods 1A). We collected active foragers on the same morning from 6 colonies, of which 3 strongly reduced foraging in dry conditions and 3 did not. For each colony, 3 replicate RNA-seq libraries were sequenced from the mRNA extracted from 3 pooled dissected forager brains. We used the kallisto/sleuth RNA-seq analysis pipeline (Bray et al., 2016) to quantify transcript expression against the *P. barbatus* reference transcriptome (Smith et al., 2011). Of the 20,387 transcripts in the reference transcriptome 273 were significantly differentially expressed in whole forager brains between the 2 sets of colonies (Figure 1A). A total of 113 transcripts were upregulated in colonies that do not reduce foraging on dry days, and 160 transcripts were upregulated in colonies that strongly reduce foraging on dry days. Across the whole transcriptome, the per-transcript mean expression levels were correlated between the 2 sets of colonies (Pearson r^2^ = 0.99). A linear principal component analysis in sleuth (Pimentel et al., 2016) showed that colony transcriptomes did not cluster clearly by behavioral type.

Overall, the list of 273 transcripts significantly differentially expressed in either direction was enriched in the terms “hormone activity,” “oxidoreductase activity,” and “copper ion binding” (p value < 0.0005, Fisher's exact test, false discovery rate [FDR] <0.25). The 160 transcripts upregulated in colonies that did not reduce foraging in dry conditions did not show any GO term enrichment with FDR < 0.9. The 113 transcripts upregulated in the colonies that strongly reduced foraging in dry conditions were enriched in GO terms “neuropeptide signaling pathway,” “catecholamine metabolic process,” and “receptor binding” (all with p value < 0.005 and FDR < 0.3). The enrichment in biogenic amine signaling and metabolism GO terms was reflected in the higher expression of the neurometabolic enzymes phenylalanine hydroxylase (3.17-fold change, XM_011648879.1, q-value = 0.0049) and tyramine β-hydroxylase (1.55-fold change, XM_011649732.1, q-value = 0.00011, alternate transcript from same locus XM_011649733.1 upregulated 1.44-fold, q-value = 1.60 × 10^−7^). The enrichment of the GO term “neuropeptide signaling pathway” was driven by increased expression of transcripts from pathways involved in the regulation of insect foraging behavior, including the FMRFamide receptor (1.69-fold change, XM_011639920.1, q-value = 0.0036), an allatostatin peptide hormone (1.2-fold change, XM_011640492.1, q-value = 1.77 × 10^−5^), and the hypertrehalosaemic prohormone (1.82-fold change, XM_011643332.1, q-value 1.60 × 10^−7^), all of which are important in insect neurohormonal signaling in solitary insects (Caers et al., 2012, Orchard and Lange, 2013, Verlinden et al., 2015). In addition, foragers from colonies that strongly reduced foraging on dry days had significantly higher expression of an inositol monophosphatase (3.1-fold change, XM_011632239.1, q-value = 5.38 × 10^−5^), a phosphoinositide phospholipase (1.33-fold change, XM_011632265.1, q-value = 0.0021), and the glycogen synthase kinase 3β interaction protein (1.54- fold change, XM_011646061.1, q-value = 0.0002). These 3 protein products are involved in the inositol phosphate signaling pathway (Berridge, 2009), implicated in the transcriptomic changes between nurse and forager honeybees (Lutz et al., 2012, Whitfield et al., 2003).

To further examine the functional relationships among brain-expressed transcripts in *P. barbatus* foragers, we performed a transcriptome-wide co-expression analysis (Mikheyev and Linksvayer, 2015, Morandin et al., 2016), using Cytoscape (see Transparent Methods 1A) (Su et al., 2014). The final transcript co-expression network consisted of 1,933 correlated transcripts across 167 isolated subnetworks. Only 2 of the 167 subnetworks had more than 50 transcripts (920 and 600 transcripts, respectively, hereafter referred to as “Module 1” and “Module 2,” Figure 1B). The 3 next-largest subnetworks had between 10 and 50 transcripts, and the remaining 162 connected subnetworks all had fewer than 6 connected transcripts. The 2 large connected transcript co-expression modules described above were biased in their mean expression values between the 2 groups of colonies. Of the 920 transcripts in Module 1, 656 (71%) had higher, but not necessarily significantly different mean expression levels in colonies that strongly reduced foraging activity in dry conditions. Of the 600 transcripts in Module 2, 545 (91%) had higher mean expression values in colonies that did not strongly reduce foraging activity in dry conditions. In addition, the modules were functionally enriched in several GO categories of neurophysiological relevance. Module 1 was enriched in GO terms “G-protein-coupled amine receptor activity,” “regulation of neurotransmitter levels,” and “postsynaptic signal transduction” (all p values < 0.001, FDR <0.05). Module 1 was strongly depleted in transcripts relating to “odorant binding” and “olfactory receptor activity.” Module 1 was significantly depleted in the GO term “cellular nitrogen compound metabolic process,” whereas this exact term was significantly enriched in Module 2. In addition, Module 2 was enriched in GO term “cellular response to stress,” and multiple GO terms relating to metabolism (all p values < 0.001, FDR < 0.05).

### Manipulation of Forager Brain Dopamine Titer Alters Foraging Activity

Based on the aforementioned transcriptomic results, we hypothesized that differences among colonies in forager brain neurophysiology could lead to colony differences in behavior. To test this hypothesis, we observed the behavior of foragers with altered brain dopamine titers in the field.

**Figure 2:**
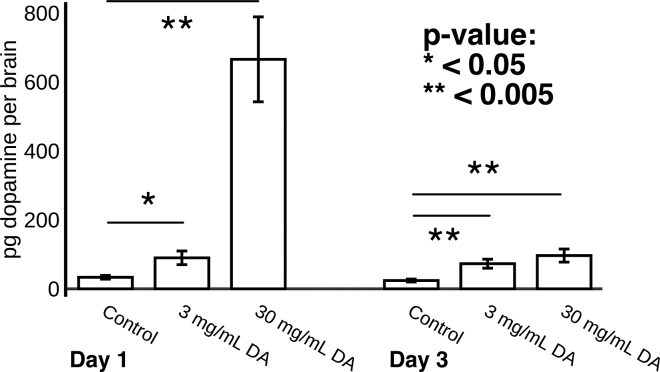
Oral Administration of Dopamine to *P. barbatus* Workers Significantly Raises Single Brain Dopamine Titers in a Dose- and Time-Dependent Fashion Data shown on y axis are mean ± SEM of dopamine titers in picograms per single dissected *P. barbatus* brain. Dopamine titer was measured 1 and 3 days after oral administration of 0 mg/mL, 3 mg/mL, and 30 mg/mL dopamine in water solution. No. of single brains measured per group is 6–9. Dopamine titer was measured by mass spectrometry with a labeled internal dopamine standard (see Transparent Methods 1B–1D).

First, we used mass spectrometry to determine that oral administration of dopamine to *P. barbatus* workers significantly raises single brain dopamine titers in a dose- and time-dependent fashion (see Transparent Methods 1B–1D). We measured single forager brain dopamine titer at 2 time points (18 and 66 hr after treatment), using two concentrations of oral dopamine solution (3 mg/mL and 30 mg/mL), with 6–9 ant brains measured per biological group. At the 18-hr time point used for later behavioral experiments, brain dopamine levels in single *P. barbatus* workers in the lower-dose (3 mg/mL dopamine) treatment group were significantly increased by 2.67-fold relative to controls (Figure 2) (t = 2.61, df = 15, p = 0.0199). In the higher-dose (30 mg/mL dopamine) treatment group, brain dopamine titers were increased by 19.91-fold relative to controls (t = 4.82, df = 15, p < 0.0002). Brain dopamine titers were still significantly increased in both treatment groups relative to controls on the third day after treatment (both p < 0.005).

**Figure 3:**
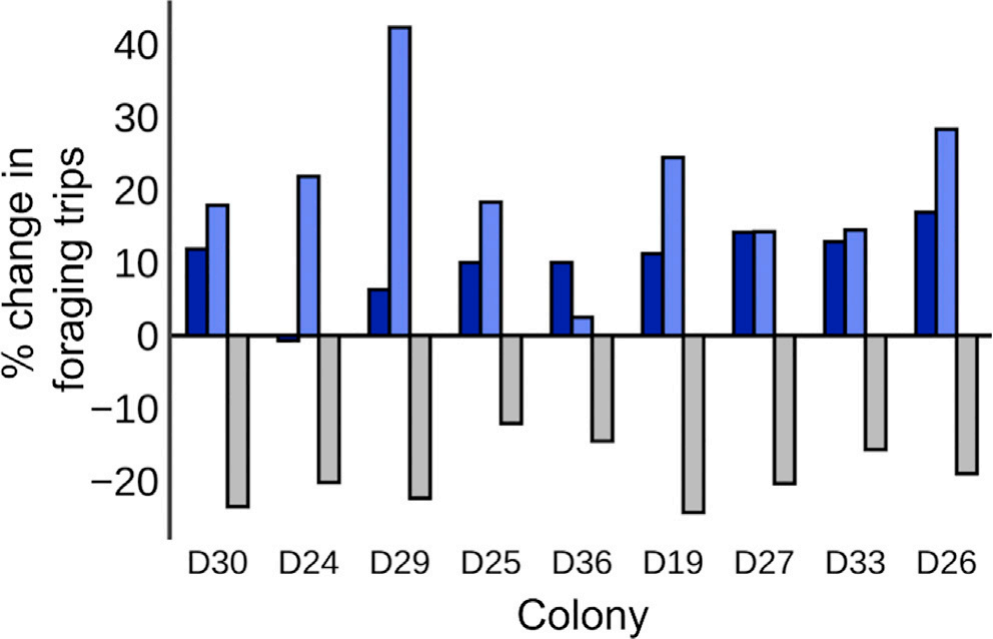
Manipulation of Forager Brain Dopamine Titer Alters Foraging Activity The x axis shows colony ID. The y axis shows the percent change in foraging trips made by drug-treated foragers relative to controls (see Transparent Methods 1E). Top bars show percent increase in foraging trips made by foragers treated with 3 mg/mL dopamine; light blue bars, 2016; dark blue bars, 2017. Bottom bars show percent decrease in foraging trips made by foragers treated with 3 mg/mL 3IY, 2017.

Next, we tested the hypothesis that increasing forager brain dopamine titer would increase foraging activity in the field (see Transparent Methods 1E). Foragers treated with 3 mg/mL dopamine made significantly more foraging trips compared with their control-treated nestmates in field experiments over 2 years (Figure 3, Data S1). In 2016, dopamine-treated foragers made on average 20.5% more foraging trips than control-treated nestmates (N = 9 colonies, effect ≠ 0, p < 0.001, t = 5.60). In the same 9 colonies in 2017, dopamine-treated foragers made on average 10.3% more foraging trips than control-treated nestmates (effect ≠ 0, p < 0.001, t = 6.09). A colony's response to dopamine in 2016 was not significantly correlated with its response to dopamine in 2017 (Kendall rank correlation p > 0.5).

To test the hypothesis that decreasing forager brain dopamine would lead to a decrease in foraging activity, we tested the effect of 3-iodotyrosine (3IY) on foraging activity. 3IY is a metabolic inhibitor that reduces brain dopamine titer in insects (Neckameyer, 1996) however, we did not use mass spectrometry to quantify brain dopamine titer change due to 3IY. In the same colonies used in the aforementioned dopamine experiments, foragers treated with 3 mg/mL 3IY made significantly fewer foraging trips than control-treated nestmates (Figure 3, Data S1). In 2017, 3IY-treated foragers made on average 19.1% fewer foraging trips than control-treated nestmates (effect ≠ 0, p < 0.001, t = 13.60).

### Colonies Naturally More Sensitive to Humidity Are More Responsive to Dopamine

Next we asked how variation in colony response to pharmacological manipulation was associated with natural variation in how strongly colonies reduced foraging activity in dry conditions. Colony reduction of foraging activity in response to humidity was quantified by estimating the decrease in daily foraging trips made by the colony per percent decrease in humidity (see Transparent Methods 1F). As in previous studies, the 9 colonies strongly differed in how much they reduced foraging activity in dry conditions. The estimated reduction in foraging trips made per colony per 1% reduction in humidity ranged from 27 to 266.

**Figure 4:**
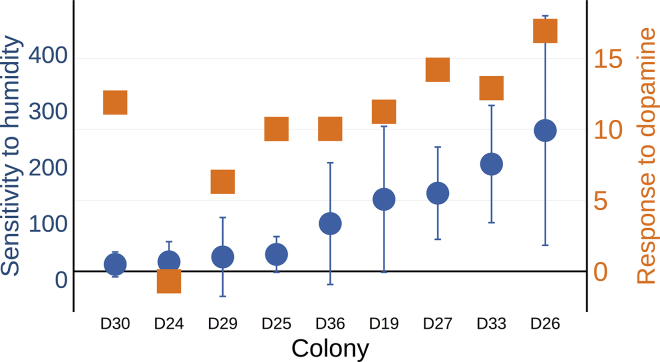
Colonies Naturally More Sensitive to Humidity Are More Responsive to Dopamine x Axis represents colony. The left y axis shows sensitivity to humidity, the estimated number of fewer foraging trips made by the colony per percent decrease in humidity (see Transparent Methods 1F). The left y axis shows sensitivity to humidity, the estimated number of fewer forager trips (± SEM) made by the colony per percent decrease in relative humidity (See Transparent Methods 1F).

Colonies that more strongly reduced foraging activity in dry conditions were more responsive to the stimulatory effect of exogenous dopamine (Figure 4) (N = 9 colonies, Kendall's rank correlation test, τ = 0.44, p = 0.013, Pearson's correlation r^2^ = 0.52, p = 0.028). There was no significant relation between a colony's response to 3IY and how strongly it reduced foraging activity in dry conditions (Pearson's correlation test p > 0.7).

### Colonies Significantly Vary in Forager Brain Biogenic Amine Content

**Figure 5:**
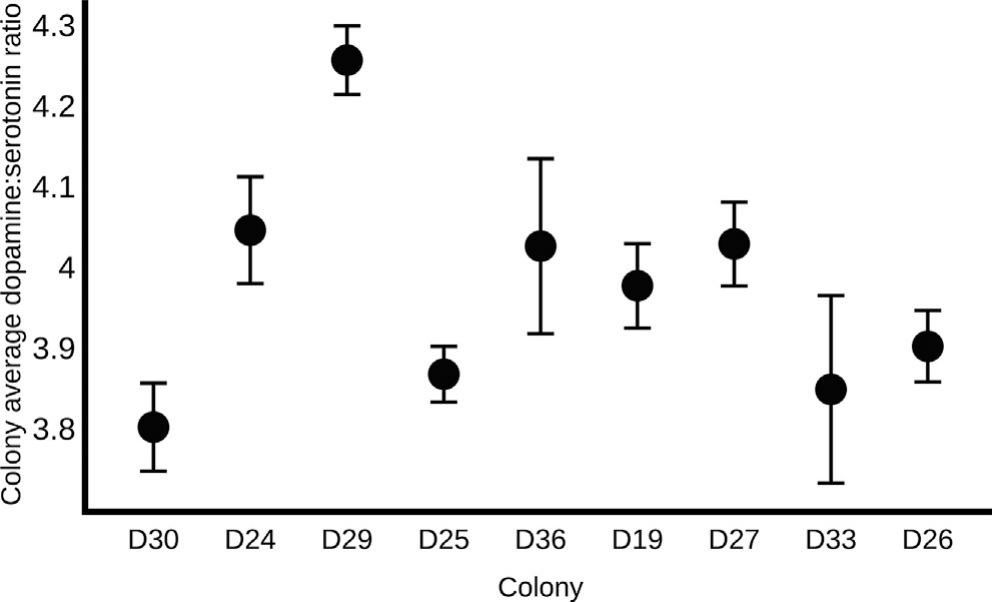
Colonies Significantly Vary in Forager Brain Biogenic Amine Content The x axis represents colony. The y axis is the average forager brain dopamine to serotonin ratio ± SEM, as measured by high-performance liquid chromatography (see Transparent Methods 1G). N = 5 samples of 2 pooled forager brains per colony.

Brain dopamine and serotonin titers were quantified from active foragers from the 9 colonies used in the aforementioned pharmacological experiments (see Transparent Methods 1G). Colonies significantly varied in their average forager brain dopamine to serotonin ratio (Figure 5) (N = 9 colonies, N = 5 samples/colony of 2 pooled brains, ANOVA for effect of colony, p < 0.001). There was no significant relationship between the colony's average forager brain dopamine to serotonin ratio and how strongly the colony reduced foraging activity in dry conditions earlier in the season (Spearman's R = −0.13, p = 0.74). Colonies with higher dopamine to serotonin ratios tended to be less responsive toward the stimulatory effects of dopamine, but this trend was not significant (Spearman's R = −0.48, p = 0.19).

## Discussion

We used transcriptomic, pharmacological, and physiological experiments to assess the molecular basis of variation among colonies of *P. barbatus* in foraging behavior.

Colonies that differed in the regulation of foraging activity significantly differed in forager brain gene expression (Figure 1A). These transcriptomic changes were enriched in biogenic amine and neurohormone-related signaling transcripts. In addition, colonies that differed in how they regulate foraging in dry conditions appeared to differ in the use of two large modules of co-expressed transcripts related to neural signaling and metabolism (Figure 1B). This suggests that differences among colonies in foraging activity may be due to differences in how foragers evaluate foraging-related stimuli, reflected in transcriptomic changes in their neural signaling pathways (e.g., as in Lucas and Sokolowski [2009]). To our knowledge, this is the first reported measurement of brain transcriptome from foraging ants in their natural context. Several of the same pathways differentially expressed between foraging and non-foraging nestmates in other social insects, such as neuropeptides and inositol phosphate metabolism (Friedman and Gordon, 2016, Kamhi and Traniello, 2013, Yan et al., 2014), were differentially expressed in the brain tissue of foragers from colonies that vary in foraging activity. These pathways are deeply conserved and often involved in regulating foraging and feeding behavior across insect species (Barron et al., 2010, Gospocic et al., 2017). Here we extend these results to suggest that the neuromolecular mechanisms involved in behavioral variation among solitary insects and social insect nestmates may also play a role in generating collective behavioral differences among colonies (Jandt et al., 2014).

Within a colony, variation among social insect workers in foraging activity has been linked to changes in the neuromodulatory biogenic amines dopamine, tyramine, and octopamine (Kamhi et al., 2017, Scheiner et al., 2006, Scheiner et al., 2017), apparently by altering the sensitivity to foraging-related cues (Kamhi and Traniello, 2013). Our transcriptomic results from forager brains, showing differential expression of the biogenic amine metabolic genes phenylalanine hydroxylase and tyramine β-hydroxylase, were consistent with alterations in either dopamine or octopamine metabolism. In studies of behavioral variation among nestmates, dopamine has consistently been associated with foraging activity in ants (Kamhi and Traniello [2013], although see Penick et al. [2014]). In addition, the role of dopamine in regulating the foraging activity of ant and bee workers has been confirmed with pharmacological experiments (Entler et al., 2016, Perry and Barron, 2013, Søvik et al., 2014), but such experiments have not yet been done to investigate the role of octopamine or tyramine in the regulation of ant foraging. Here, we tested only the role of dopamine, and further work is needed to examine the role of other biogenic amines in the regulation of foraging in harvester ants.

We found differential expression of key biogenic amine metabolic loci in forager brain tissue from colonies that differed in their sensitivity to humidity. In addition, biogenic amine metabolism-related transcripts were significantly enriched in the list of transcripts upregulated in colonies sensitive to humidity. This suggests that colony differences in sensitivity to humidity may be related to differences in forager brain biogenic amine metabolism, although transcriptomic differences alone are not sufficient to demonstrate physiological impact. Biogenic amine metabolic loci have a well-known role in insect cuticle sclerotization and tanning (Kramer et al., 2001, Rebeiz and Williams, 2017) and may influence desiccation tolerance or coloration in *P. barbatus*. However, our RNA-seq data were generated from dissected brain tissue without residual head cuticle, so we cannot determine whether forager cuticle expression of biogenic amine metabolic loci is associated with variation among colonies in foraging behavior. Whole-brain biogenic amine titers vary consistently between foraging and non-foraging workers in ant and bee colonies (Kamhi and Traniello, 2013), but previous transcriptomic studies have not identified differential expression of biogenic amine metabolic genes between nestmates (Feldmeyer et al., 2014, Manfredini et al., 2014, Mikheyev and Linksvayer, 2015). This may be because brain-specific transcriptomic changes important for biogenic amine metabolism are obscured when whole-body or whole-head gene expression profiles are measured (Johnson et al., 2013). Alternatively, associations between worker task and brain biogenic amine content may be driven by mechanisms other than the brain-specific differential expression of metabolic loci, for example, by changes in metabolite transport from the hemolymph.

Pharmacological increases of forager brain dopamine significantly increased foraging activity in foragers relative to their nestmates the following day (Figure 3). Conversely, ostensible reductions of forager brain dopamine significantly reduced foraging activity the following day (Figure 3). To our knowledge this is the first behavioral pharmacological manipulation of biogenic amine neurophysiology in ants in the field. These experiments link dopamine signaling and foraging activity in ants, showing a positive association that is consistent with the results of previous pharmacological studies on the role of dopamine signaling in the regulation of insect foraging (Perry and Barron, 2013, Søvik et al., 2015a).

There are several non-exclusive behavioral mechanisms that may explain how changes in dopamine signaling influence an individual forager's decision to leave the nest on its next trip. First, changes in dopamine signaling could change how a forager perceives interactions with nestmates. Previous work has suggested that increases in brain dopamine titer increase an ant's sensitivity to foraging cues such as pheromone trails (Kamhi and Traniello, 2013). Foragers of *P. barbatus* are not stimulated to leave the nest by pheromone trails (Prabhakar et al., 2012). Instead, olfactory interactions between outgoing and returning foragers with food stimulate outgoing foragers to leave the nest (Davidson et al., 2016, Pinter-Wollman et al., 2013). We suggest that increases in dopamine signaling may increase forager sensitivity to these olfactory interactions, whereas decreases in dopamine signaling may decrease forager sensitivity to interactions. In this way, increased dopamine signaling might override the negative influence of low humidity. Second, changes in dopamine signaling could alter the forager's perception of its own physiological state or the harshness of the environment, including low humidity. Self-evaluation of physiological state is important in the regulation of individual foraging activity in other ant species (Robinson et al., 2012, Silberman et al., 2016), and dopamine can influence the evaluation of environmental stimuli and organismal state (Barron et al., 2015, Friston et al., 2012, Scaplen and Kaun, 2016). Thus, increases in dopamine signaling may lead dopamine-treated *P. barbatus* foragers to overestimate their physiological readiness for foraging given the perceived humidity, and vice-versa for 3IY-treated foragers. Third, dopamine signaling influences the light-dependent circadian rhythm of insects (Hirsh et al., 2010, Nall and Sehgal, 2014), and changes in dopaminergic signaling could interact with circadian patterns of gene expression in the brain of *P. barbatus* foragers (Ingram et al., 2005, Ingram et al., 2016) and stimulate foraging despite low humidity. Finally, our pharmacological treatments may alter foraging activity by changing the brain titer of some neurotransmitter other than dopamine. By the time of the behavioral observation, some of the ingested dopamine may have been metabolized into related compounds such as tyramine or octopamine. Both tyramine and octopamine regulate some aspects of foraging in bees (Kamhi et al., 2017, Scheiner et al., 2006, Scheiner et al., 2017), although less is known about their role in ant foraging. Similarly, 3IY may modulate the brain titers of biogenic amines other than dopamine, or act directly on aminergic receptors.

Colonies that more strongly reduced foraging activity in dry conditions were more sensitive to the stimulatory effects of dopamine on foraging activity (Figure 4). This significant correlation suggests that exogenous dopamine may improve forager perception of daily conditions, generating a more positive response to the cues that stimulate foraging in the colonies that reduce foraging activity most when conditions are poor. Alternatively, elevated forager brain dopamine titers may simply override the forager's ability to detect that environmental conditions are poor, eliciting a higher stimulatory response in colonies most sensitive to changes in ambient humidity. Variation among colonies could arise from shared genetic or epigenetic factors that influence dopaminergic neurophysiology. Colonies of *P. barbatus* may show stable differences in how they regulate foraging activity in dry conditions (Gordon, 2013) due to persistent factors that modulate the influence of humidity on forager behavior.

Colonies significantly differed in average forager brain dopamine to serotonin ratio (Figure 5). To our knowledge, this is the first measurement of brain biogenic amine titers in an ant species outside of the laboratory. Differences among colonies in forager brain biogenic content were not correlated with variation among colonies in sensitivity to humidity or response to pharmacology. Thus variation among colonies of *P. barbatus* in foraging activity may be due to changes in neurophysiology at a finer scale than the whole brain. For example, biogenic amine metabolic differences between foraging and non-foraging honeybees occur within specific subregions (Schulz et al., 2003). The expression of dopamine receptors or dopamine-activated neural signaling pathways may also influence behaviorally important dopaminergic neurophysiology (Landayan et al., 2018, Yamamoto and Seto, 2014). Such non-metabolic effects in biogenic amine signaling pathways would not lead to observable differences among colonies in their average level of forager whole-brain dopamine to serotonin ratio. Our transcriptomic results from forager brains showed that differences between colonies in foraging behavior are associated with the expression of genes related to neural signaling aspects of biogenic aminergic neurophysiology. Forager brain octopamine or tyramine titers may also be important in foraging activity (Kamhi et al., 2017, Perry and Barron, 2013, Scheiner et al., 2017) and were not measured here. Further analyses of forager neurophysiology are needed to explore how differences among colonies in brain biogenic amine signaling and metabolism are associated with differences among colonies in behavior.

Here we link variation among colonies in sensitivity to humidity with variation among colonies in forager dopaminergic neurophysiology. Transcriptomic results suggested that natural variation among colonies in forager neurophysiology may be the source of differences among colonies in sensitivity to humidity, implicating biogenic amine and neurohormonal signaling. Pharmacological experiments found that foragers from colonies who reduced foraging most in dry conditions were most stimulated by exogenous dopamine. This further supports a role for dopamine signaling in the variation among colonies in sensitivity to humidity.

Our study leaves open several questions to be pursued in future research. First, our transcriptomic results implicate a variety of molecular pathways that are associated with behavioral differences among colonies. The transcriptomic differences were enriched in loci related to biogenic amine metabolism and signaling. This result is consistent with a role not only for dopamine in behavioral differences among colonies but also for other neuromodulators such as tyramine or octopamine. Although our pharmacological treatments demonstrated a clear influence on foraging behavior of altered brain dopamine titers, it would be interesting to measure the influence of drug treatments on the metabolism of biogenic amines other than dopamine, or on other neurotransmitter receptors. Second, our results do not specify whether the increase in foraging activity due to dopamine treatment is because a few foragers dramatically increase foraging activity, or whether most increase activity slightly. We are currently investigating this in experiments with individually marked ants. Finally, there is much to learn about how dopamine affects a forager's decision to leave the nest on its next foraging trip and how this is related to the stimulation of foraging by the rate of olfactory encounters with returning foragers (Davidson et al., 2016, Pinter-Wollman et al., 2013).

Molecular studies in social insects have primarily examined differences between workers performing different tasks within the same colony (Johnson and Linksvayer, 2010, Kamhi and Traniello, 2013, Linksvayer, 2015, Robinson, 2003). However, it is the variation among colonies in task performance that leads to variation in reproductive success and thus the evolution of collective behavior (Gordon, 2011, Gordon, 2013, Gordon, 2016b). To understand the evolution of colony behavior, we need to learn how patterns of molecular variation among colonies are shaped by the interaction of genetic, epigenetic, and environmental factors (Abouheif et al., 2014, Rehan and Toth, 2015, Toth and Rehan, 2017, Toth and Robinson, 2007, West-Eberhard, 2003).

## Methods

All methods can be found in the accompanying Transparent Methods supplemental file.

## Data and Software Availabilty

Reads are available in the Short Read Archive (BioProject: PRJNA277638).

## Supplemental Information

Document S1. Transparent Methods and Table S1

Data S1. Pharmacology Raw Data, Related to Figure 3
