# Supplementary material for: The Role of Dopamine in the Collective Regulation of Foraging in Harvester Ants
Source: iScience. 2018 Sep 27;8:283–94. doi: 10.1016/j.isci.2018.09.001 (PMC6205345; doi:10.1016/j.isci.2018.09.001)
Supplement: Document S1. Transparent Methods and Table S1 [file mmc1.pdf]

ISCI, Volume *8*

Supplemental Information

The Role of Dopamine in the Collective Regulation of Foraging in Harvester Ants

Daniel A. Friedman, Anna Pilko, Dorota Skowronska-Krawczyk, Karolina Krasinska, Jacqueline W. Parker, Jay Hirsh, and Deborah M. Gordon

Supplemental Information

1. Transparent Methods

A. Transcriptomic Methods
B. Pharmacology Methods
C. Laboratory experiments on the effect of dopamine administration
D. Mass spectrometry methods
E. Field pharmacology experiments
F. Behavioral ecological methods
G. Brain Biogenic Amine Content.
2. Table S1. Mass Spectrometry information for analyte detection, Related to Figure 2
3. Data S1. Pharmacology Raw Data, Related to Figure 3.
4. Supplemental References

---

1. Transparent Methods

A. Transcriptomic Methods

Foragers of *Pogonomyrmex barbatus* were collected into liquid nitrogen between 06:00-08:00 on 8/20/2014, from colonies at a long-term field site near Rodeo NM, at which all colonies have been identified and censused since 1985 (Ingram et al., 2013). Foragers were collected as soon as they left the nest entrance, not carrying anything, and moved off the nest mound onto a foraging trail or fan. Foragers were collected from 6 mature colonies in which foraging behavior had been monitored in previous work. Three of the colonies strongly reduced foraging on dry days relative to humid days, in counts of foraging activity made in in 2011 and 2012 (Gordon, 2013), while the other 3 colonies did not strongly reduce foraging activity on dry days. There were similar differences between some of the colonies in each group in foraging activity measured in 2009 (Gordon et al., 2011). Other work shows that colonies are consistent from year to year in foraging activity (Gordon, 1991; Gordon et al., 2013). No ethical precautions were required for this study.

Samples were shipped from the field site to the laboratory in liquid nitrogen (Cryoship), and stored at -80C. Whole brains were cleanly dissected away from muscular, glandular, and connective tissue in cold RNAlater buffer. Dissected brains were frozen in Triazol at -80C until RNA extraction. Total RNA was extracted from dissected brains using a Direct-zol RNA extraction kit (Zymo Research). RNA concentration was assessed using Qubit 2.0 RNA HS reagents (Thermo Fisher Scientific) and purity using a NanoDrop (ND 2000, Thermo Scientific). Total RNA was assessed for quality using a BioAnalyzer tapestation (Agilent Technologies), and samples with RNA Integrity Number (RIN) > 8.0 were used to make RNA libraries. 3 libraries were made for each of 6 colonies. Each library consisted of poly-AAA+ mRNA extracted from the pooled dissected brains of 3 foragers. Libraries were generated using Illumina's TruSeq Stranded mRNA Sample Prep Kit. Reads are available in the Short Read Archive (BioProject: PRJNA277638).

For the kallisto/sleuth differential gene expression analysis pipeline, the reference transcriptome was indexed by kallisto v.0.42.5 (Bray et al., 2016). RNA-seq reads were pseudoaligned to the indexed reference transcriptome with kallisto, a method with good consistency to other RNA-seq and rt-qPCR quantifications of differential expression (Costa-Silva et al., 2017). The k-mer bias correction option was implemented and 100 bootstrapped transcriptomes were generated for each library to estimate the variation of expression for each transcript. The kallisto output was analyzed using the sleuth v0.28.0 package (Pimentel et al., 2016) in R v.3.3.0 (R Development Core Team, 2014). Across the 18 libraries from 6 colonies, there were a total of ~355 million 75 basepair paired-end RNA-seq reads. All workers sampled in this study are part of the same interbreeding J1/J2 population of *P. barbatus* at a long-term study site (Ingram et al., 2013) and no colonies or libraries displayed a mapping bias to the reference transcriptome used. A post-correction q-value threshold of 0.01 was used to call a transcript as differentially-expressed.To generate a transcript co-expression network, the "ExpressionCorrelation" plugin was used within Cytoscape (Su et al., 2014) with a cutoff of transcript-transcript Pearson correlation $r^2 > 0.93$ across all 18 libraries.

To generate functional annotations of the reference transcriptome, InterProScan (Jones et al., 2014) was used to query each transcript's predicted protein translation against 11 protein databases (Profile HMM models: CATH-Gene3D, Superfamily, PIRSF, TIGRFAMs, Panther, Pfam, and Smart. Profile models: HAMAP, Prosite, ProDom. Pattern models: PRINTS, Prosite). InterProScan protein domain-level GO terms were merged with the GO terms inferred by blastx homology in Blast2GO. Annotation augmentation (ANNEX) was performed in Blast2GO. Lists of transcripts identified from differential expression co-expression analyses were tested for GO term enrichment using Fisher's Exact Test in Blast2GO. Multiple test correction was implemented according to the False Discovery Rate (Benjamini and Hochberg, 1995). Enriched or depleted GO terms with three or fewer annotated representatives in a given gene set were not considered.

B. Pharmacology Methods

All solutions were administered to ants as follows: an ant was collected with an aspirator and placed in a 50 mL tube. The 50 mL tube was immersed in ice until the ant stopped moving. The ant was tapped out onto a paper towel, and gently grasped by a rear leg. To mark the ant, a small dab of oil-based paint (Uni-Paint PX-20) was placed on the back of the ant's head using a small toothpick, using a unique color for each of the treatment groups. To feed the solution to the ant, 0.2 µL of aqueous solution was placed on the mandibles of the anesthetized ant. The droplet is captured between the mandibles via surface tension. The contents of the solution used in each experiment are described for each experiment specifically. Where applicable, all drug solutions were prepared fresh from powder and measured with a Mettler Toledo AT261 Delta Range FACT to 0.1 mg accuracy. After administering a solution to an ant, the ant was placed on its lateral side, and it eventually began to move around

C. Laboratory experiments on the effect of dopamine administration.

Foraging *Pogonomyrmex barbatus* were collected from adult colonies near Phoenix, AZ in 5/2016, and driven to Stanford, CA. Laboratory ants were kept on a 14:10 LD cycle in a temperature controlled room (74 degrees F). Ants were given ad libitum access to wet cotton balls inside of glass tubes for water, and provided apple slices and millet seed every 3 days. Ant nests consisted of a foraging arena (2' x 3') with Fluon-coated sides (BioQuip) that had an open top and was exposed to light, connected to a series of smaller plastic boxes (4" x 4" ranging to 8" x 12") that were kept in the dark. Ants for brain dopamine quantification were collected from the foraging arena of the laboratory colony. All collected ants were placed into the same container, then randomly sorted into three treatment groups.

For treatment, ants were individually slowed on ice then orally administered either pure water (control), water with 3 mg/mL dopamine (3.714 mg/mL dopamine hydrochloride salt) (Sigma-Aldrich, PubChem 24277897), or water with 30 mg/mL dopamine. The solubility of dopamine in water is 600 g/L (PubChem CID: 681) and all dopamine solutions were made immediately before administration. After treatment, ants were kept in laboratory conditions in a fluon-coated plastic box. Ants were collected the following morning at 9am (day 1 time point) or 3 days after ingestion at 9am (day 3 time point). At the time of collection, ants were flash frozen in liquid nitrogen and stored at -80C until dissection. The brain was refrozen in 50 µL PBS on dry ice, then stored at -80C until dopamine quantification was performed at the Stanford University Mass Spectrometry facility with an internal radiolabeled dopamine standard as follows.

D. Mass spectrometry methods:

Brain samples containing one ant brain and 50 uL PBS were placed on ice in 1.7 mL tubes and 5 µL of 5 µM *d4*-dopamine (dopamine internal standard, Cambridge Isotope Laboratories) solution was added followed by 60 µL of 4% formic acid. Samples were vigorously pipetted for 60 sec to start the homogenization. After that samples were placed in ice water bath and sonicated for 10 minutes twice. Next 400 µL of 0.1 % formic acid in cold acetonitrile was added to the sample and sonicated for 10 more minutes. Samples were then centrifuge for 10 min @ 4°C @ 14000 rpm, dried under nitrogen and reconstituted in 50 µL of HPLC sample buffer (2 mM ammonium formate pH 3.2 / 20% Methanol). The separation of derivatized amino acids was performed on an HP1100 HPLC system (Agilent, Santa Clara, CA) using a 150 x 2.10 mm Luna-PFP column (Phenomenex, Torrance, CA) after injection of 5 µL at a flow rate of 0.3 mL/min. Mobile phase A was 2 mM ammonium formate pH 3 in water. Mobile phase B was 2 mM ammonium formate in methanol. The gradient program was as follows: 0.00-2.00 min – 20%B, 4.00 min – 80% B, 5 min – 80% B, 6-8 min – 20% B. All samples were injected in triplicates. A Quattro Premier triple quadrupole mass spectrometer (Waters, Milford MA) was operated in positive electrospray ionization mode. Capillary voltage was set to 3.00 kV, and cone voltage was set to 21 V. The detection of the analytes was performed in selected reaction monitoring mode (SRM). Two or three precursor ion - fragment ion pairs were selected for each analyte and compound specific cone voltage and collision energy values were used (see Table S1). Stable isotope labeled *d4*-dopamine was utilized as an Internal Standard (IS). The most intense transition for each analyte was selected for quantitation and the subsequent ones for confirmation.

An 8-point dopamine calibration curve was used with all analytes ranging from 2nM to 4000nM, and *d4*-dopamine at fixed 5 nmol/mL concentration as an internal standard. The LLOD for dopamine was 50 fmol on column and LLOQ was 150 fmol.

E. Field pharmacology experiments

Behavioral pharmacological experiments were performed with a set of 10 colonies in 7-8/2016 (D19, D24, D25, D26, D27, D29, D30, D33, D34, D36), and in 9 of the same 10 colonies in 8/2017 (all the previous colonies except D34). The colonies were near but not on the long-term study site (Ingram et al., 2013). Foragers were collected 1-2 meters from the nest entrance and identified as foragers because they were not carrying anything and walked in a straight line off the nest mound towards a foraging trail or fan (Gordon, 1986). The ants were brought back to the laboratory at the Southwestern Research Station and randomly sorted into treatment groups, defined as follows. In 2016, there were two treatment groups: 1x phosphate-buffered saline (PBS, Electron Microscopy Sciences), and 3 mg/mL dopamine in 1x PBS group. In 2017, there were three treatment groups: 1x PBS, 3 mg/mL dopamine in 1x, and 3 mg/mL 3-iodo-tyrosine (3IY, Sigma-Aldrich). In both years, each treatment group consisted of 100-150 foragers per colony, the same number of foragers was used between groups for each colony replicate. Ants were returned to their nest mound later the same day and most returned marked ants immediately descend into their nest. Foragers of *P. barbatus* tend to be the oldest ants in the colony, and workers marked while foraging do not later switch to perform other tasks (Gordon, 1989).

Observations began early the following day before foraging began. Counts of foraging trips by marked ants began when the first marked ant was observed to leave the nest. For colonies with a single foraging trail, a foraging trip was recorded when a marked ant crossed a line ~1-2 meters from the nest entrance on the trail. For colonies with more than one foraging trail, a foraging trip was recorded when a marked ant was observed leaving the nest entrance, carrying nothing, and walking in a straight line off of the nest mound (Gordon, 1986). In 2016, two colonies were observed each morning in alternating observation periods of 15-20 minutes. During each 15-20 minute observation period, all foraging trips made by marked ants were marked and counts were recorded in 30-second intervals. Foraging counts ended when the colony had stopped foraging for the morning and no ants had left the nest for 3 minutes. In 2017, one colony was observed per day, and outgoing foraging trips of marked ants were counted in 30-second intervals as before.

For each colony we calculated the response to drug treatment as the increase in foraging trips made by dopamine- or 3IY-treated ants divided by the total number of foraging trips made by control-treated foragers. This design minimizes the effects of day, as all comparisons are being made between two groups of foragers within the same colony on the same day. In each interval the numbers of foraging trips made by workers from each treatment were not very different and the results in each interval did not always reflect the totals: in only about one-third of the 30-second intervals were there were more dopamine-treated foragers than PBS-treated nestmates.

F. Behavioral ecological methods

Observation of undisturbed colony foraging behavior occurred during August 2017. Observation was performed over 12 consecutive days (8/3/17 - 8/14/17) on the set of 9 colonies later used in pharmacological experiments. Each colony was surrounded with 5 field flags, each placed 1.5 meters from the nest entrance, with the first flag pointing due North. On observation days, colonies were observed in a circuit by the same observer, in an order that was altered every day. Observation began before any colony had begun foraging for the day, at around 5am. If colonies were not foraging at all at the time of observation, then the observer proceeded to the next colony immediately. An outgoing foraging trip was defined as occurring when an unladen ant walked in a straight line off the nest mound and crossed an invisible line between two of the field flags surrounding the colony. An incoming foraging trip was defined as occurring when an ant crossed an invisible line between two field flags, heading towards the nest entrance. If more than 1 incoming and/or outgoing foragers were observed in the first 30 second scan per each side of the colony, rates of incoming and outgoing foragers were counted in 3 sequential 30-second intervals for that side. Observation of focal colonies continued until all colonies had finished foraging for the day. Average incoming and outgoing foraging rates per colony per observation were calculated as follows. First, the average incoming and outgoing foraging rate per minute per side was calculated by doubling the average of the 3 sequential 30-second counts per side. Then, the colony overall foraging rate per minute was calculated for each observation by summing foraging rates across all 5 sides of the colony.

The estimated overall number of foraging trips made by each colony each day was calculated as the area under the curve of colony overall outgoing foraging rate through time, as the integral of outgoing foraging rate through time is the overall number of foraging trips. The “weatherData” package was used to obtain weather data for all foraging days from a climate station near the fieldsite in Rodeo, NM. For each day where undisturbed colony behavior was observed, the average relative humidity measurement between 07:00 and 12:00 was calculated. Parametric ANOVA modeling was used to test for differences among colonies in how their total number of trips per day was associated with the daily humidity. To quantify the sensitivity of each colony to humidity, the total number of foraging trips made by the colony per day was regressed using a Theil-Sen non-parametric estimator (R “mblm” package) against the average relative humidity that day. The slope of this regression represents the estimated number fewer total foraging trips made by the colony per percent decrease in relative humidity, where higher values reflect higher colony sensitivity to humidity.

To test for a relationship between colony sensitivity to humidity and response to pharmacology, the value of the regression slope estimated above was correlated with the percent increase in foraging trips made by dopamine-treated ants relative to control-treated ants from that colony. Both parametric Pearson and non-parametric Kendall correlation tests were performed and correlation estimates are reported in the main text.

G. Brain biogenic amine content methods.

Natural variation in biogenic amine titer among foraging ants from colonies of *P. barbatus* was measured with High-Performance Liquid Chromatography (HPLC).

Foragers from the 9 focal colonies were collected on the morning of 9/4/2017 between 7am and 9am. Collected ants were frozen directly into liquid nitrogen, and kept in liquid nitrogen or a -80C freezer until dissection in cold citric acid. For HPLC we measured 5 samples from each of the 9 colonies with collected ants. Each sample consisted of 2 pooled brains and was measured for dopamine and serotonin content as per Hardie and Hirsh (Hardie and Hirsh, 2006).

Statistical analysis of natural variation among colonies in forager brain neurotransmitter titer was performed in R v3.4.0 with an ANOVA test from the library "heplots". The library "granovaGG" was used to visualize the ANOVA results.

2. Table S1. Mass Spectrometry information for analyte detection, Related to Figure 2

Analyte	Abbreviation	SRM transitions	Cone Voltage [V]	Collision Energy [eV]
Dopamine	DA	154.0 > 90.7<br>154.0 > 118.7<br>154.0 > 136.7	17<br>17<br>17	21<br>17<br>11
Dopamine IS	DA IS	158.0 > 121.7<br>158.0 > 140.8	17<br>17	21<br>11
Histamine	HA	111.8 > 67.6<br>111.8 > 94.6	22<br>22	19<br>13
Tyrosine	TYR	138.0 > 76.7<br>138.0 > 94.6<br>138.0 > 102.7	15<br>15<br>15	25<br>16<br>20
Norepinephrine	NOREPI	170.0 > 106.7<br>170.0 > 134.7<br>170.0 > 151.8	11<br>11<br>11	22<br>16<br>7
Serotonin	SRT	176.9 > 114.6<br>176.9 > 131.8<br>176.9 > 159.7	18<br>18<br>18	28<br>22<br>12
Epinephrine	EPI	184.0 > 107.0<br>184.0 > 134.9<br>184.0 > 166.0	15<br>15<br>15	22<br>15<br>9

3. Data S1. Pharmacology Raw Data, Related to Figure 3.

File attached as supplemental dataset.

4. Supplemental References.

Benjamini Y and Hochberg Y (1995) Controlling the False Discovery Rate: A Practical and Powerful Approach to Multiple Testing. *J. R. Stat. Soc. Series B Stat. Methodol.* 57(1): 289–300.

Bray NL, Pimentel H, Melsted P, and Pachter L (2016). Near-optimal probabilistic RNA-seq quantification. *Nat. Biotechnol.* 34: 525–527.

Costa-Silva J, Domingues D, and Lopes FM (2017). RNA-Seq differential expression analysis: An extended review and a software tool. *PLoS One* 12, e0190152.

Gordon DM (2013). The rewards of restraint in the collective regulation of foraging by harvester ant colonies. *Nature* 498: 91–93.

Gordon DM (1991). Behavioral Flexibility and the Foraging Ecology of Seed-Eating Ants. *Am. Nat.* 138(2): 379–411.

Gordon DM (1986). The dynamics of the daily round of the harvester ant colony (*Pogonomyrmex barbatus*). *Anim. Behav.* 34(5): 1402–1419.

Gordon DM (1989). Dynamics of task switching in harvester ants. *Anim. Behav.* 38(2): 194–204.

Gordon DM, Dektar KN, and Pinter-Wollman N (2013). Harvester ant colony variation in foraging activity and response to humidity. *PLoS One* 8, e63363.

Gordon DM, Guetz A, Greene MJ, and Holmes S (2011). Colony variation in the collective regulation of foraging by harvester ants. *Behav. Ecol*. 22(2): 429–435.

Hardie SL and Hirsh J (2006). An improved method for the separation and detection of biogenic amines in adult *Drosophila* brain extracts by high performance liquid chromatography. *J. Neurosci. Methods* 153(2): 243–249.

Ingram KK, Pilko A, Heer J and Gordon DM (2013). Colony life history and lifetime reproductive success of red harvester ant colonies. *J. Anim. Ecol.* 82(3): 540–550.

Jones P, Binns D, Chang H-Y, Fraser M, et al. (2014). InterProScan 5: genome-scale protein function classification. *Bioinformatics* 30(9): 1236–1240.

Pimentel HJ, Bray N, Puente S, Melsted P, and Pachter L, (2016). Differential analysis of RNA-Seq incorporating quantification uncertainty. *bioRxiv*. https://doi.org/10.1101/058164

R Core Team (2014). R: A language and environment for statistical computing. R Foundation for Statistical Computing, Vienna, Austria. URL http://www.R-project.org/.

Su G, Morris JH, Demchak B, and Bader GD (2014). Biological network exploration with Cytoscape 3. *Curr. Protoc. Bioinformatics* 47: 8.13.1–8.13.24.
